# SADRP: A structurally aware multi-modal framework for drug response prediction

**DOI:** 10.1371/journal.pcbi.1014861

**Published:** 2026-10-21

**Authors:** Ren Qi, Shujia Liu, Tianhong Quan, Tianru Wu, Alexey K. Shaytan, Bin Liu

**Affiliations:** 1 School of Computer Science and Technology, Beijing Institute of Technology, Beijing, China; 2 Zhongguancun Academy, Beijing, China; 3 Department of Biology, Lomonosov Moscow State University, Moscow, Russia; 4 SMBU-MSU-BIT Joint Laboratory on Bioinformatics and Engineering Biology, Shenzhen MSU-BIT University, Shenzhen, Guangdong, China; Humanitas University, ITALY

## Abstract

**Motivation:**

Predicting drug sensitivity across diverse cancer samples is a fundamental challenge in personalized therapy and drug discovery. This task requires comprehensive characterization of both drug and cancer cell line features. Current approaches often fail to fully capture the complex structural characteristics and interactions of drug compounds and struggle to integrate multi-omics features derived from the same sample, limiting their feature representation capacity. Consequently, there is a need for modeling frameworks that can jointly model molecular structural features and multi-modal cellular contexts in a unified manner.

**Results:**

We present SADRP, a structurally aware multi-modal framework for drug response prediction, designed to model complex molecular structures and multi-omics data. For drug representation, SADRP incorporates Shortest Distance Path (SDP) encoding and edge encoding to capture both topological relationships and bond-type chemical semantics. For cell line profiling, a multi-view interactive encoder integrates gene expression and DNA methylation, capturing cross-omics synergistic complementarity. SADRP consistently outperforms state-of-the-art methods, while also identifying potential therapeutic candidates and biomarkers, providing mechanistic insights and supporting applications in drug discovery and personalized medicine.

## Author summary

Cancer patients often respond differently to the same anticancer drug, making effective treatment selection a major challenge in precision oncology. Although computational models have been widely developed for drug response prediction, many existing approaches remain limited in their ability to accurately represent complex drug structures and integrate multiple layers of cellular information. To address these challenges, we developed SADRP, a structurally aware deep learning framework that models both the topological relationships and chemical bond semantics of drug molecules while jointly integrating gene expression and DNA methylation profiles from cancer cell lines. By combining these complementary sources of information, SADRP provides more comprehensive representations of both drugs and cellular states. We evaluated SADRP on large-scale benchmark datasets and found that it consistently outperformed several state-of-the-art methods. In addition to improving prediction accuracy, SADRP identified potential therapeutic candidates and biologically relevant biomarkers associated with drug sensitivity. Our results demonstrate the value of integrating molecular structural information with multi-omics data to support drug discovery and precision cancer therapy.

## 1 Introduction

Drug response prediction (DRP) plays a pivotal role in modern pharmacology and precision medicine, aiming to anticipate the effects of various compounds on biological systems, thereby guiding personalized therapeutic strategies [1]. In oncology, DRP is particularly challenging due to pronounced biological heterogeneity, as illustrated by cancer, where drug efficacy can vary substantially across tumor subtypes and individual patients [2,3]. Such intrinsic variability poses significant challenges for conventional experimental or statistical modeling of drug responses, underscoring the critical role of computational and AI-based approaches that leverage large-scale drug response datasets to uncover underlying response patterns [4–10].

Early drug response prediction studies primarily relied on statistical tests to identify single-gene biomarkers, such as evaluating whether patients harboring an EGFR mutation exhibit increased sensitivity to gefitinib [11]. Subsequent machine learning methods incorporated drug descriptors and multi-omics features to predict drug response [12–14], making data-driven prediction of drug sensitivity feasible. However, conventional machine learning methods often struggle with high-dimensional omics data in the presence of limited cell line samples, which increases the risk of overfitting and limits model generalization [15,16]. In addition, their reliance on fixed-length binary chemical fingerprints limits the ability to capture rich molecular topology and structural context beyond predefined substructures [17]. Driven by the establishment of large-scale drug response datasets and breakthroughs in neural architectures, deep learning-based drug response prediction frameworks have emerged, typically characterized by an end-to-end architecture comprising three modules: a drug feature encoder, a cell line encoder and a response predictor [18–21]. The drug encoder captures drug features from representations such as SMILES sequences, molecular graphs, or fingerprints [22–24], while the cell line encoder extracts cell line representations from multi-omics data, including gene expression and DNA methylation [25–27]. These latent representations are then integrated to predict drug efficacy, often measured by IC50 values or binary sensitivity labels.

In the early development of deep learning-based DRP, CDRscan [28] pioneered the use of deep neural networks in this domain, while subsequent models such as tCNNs [29] and MOLI [30] primarily relied on Convolutional Neural Networks (CNNs) [31] to extract features from chemical descriptors and genomic mutations. However, CNNs are limited in capturing the topological relationships and local chemical environments inherent in molecular graphs, which has motivated researchers to adopt Graph Neural Networks (GNNs) [32] for more effective modeling of molecular structures. Transitioning into the current era, most approaches adopt similar designs for cell line encoders and response prediction. However, they differ substantially in how the drug encoder is designed. DeepCDR [33], DeepDRP [34], and MolMVC [35] represent drug molecules as graphs and utilize GNNs to extract topological features, whereas DeepTTA [36] treats drugs as SMILES sequences and leverages Transformer [37] architectures for representation learning. More recently, hybrid graph transformer-based architectures, such as DeepAEG [38] and MCMVDRP [39], have been developed to integrate GNN-based message passing with Transformer attention, aiming to capture both local chemical motifs and long-range molecular dependencies.

Despite considerable progress, current approaches remain limited in handling complex molecular representations and high-dimensional omics data. On the drug side, although GNNs are effective in capturing local structural patterns, they often fail to resolve global molecular contexts due to message-passing constraints and frequently neglect the rich chemical semantics embedded in edge-level bond features [40,41]. While adept at modeling long-range dependencies, sequence-based Transformers completely neglect molecular geometry and suffer from the absence of pre-trained representations, unlike their successful counterparts in natural language processing [42]. Even hybrid graph transformer-based models provide limited spatial characterization and still fail to incorporate edge-level attributes, limiting their ability to resolve complex molecular representations [43,44]. On the cell line side, most studies process multi-omics data in isolation, combining them only through late fusion, which fails to resolve the mutual complementarity between cross-omics features valuable for modeling complex cellular responses.

To address these limitations, we propose SADRP, a **S**tructurally **A**ware multi-modal framework for **D**rug **R**esponse **P**rediction. For drug representation, SADRP enhances the graph transformer architecture with two key structural encodings: Shortest Distance Path (SDP) encoding and edge encoding [45]. Specifically, SDP encoding embeds shortest path distances between atoms to provide the topological awareness necessary for accurate spatial characterization. Furthermore, edge encoding is employed to incorporate bond-type semantics, overcoming the limited edge-level modeling that constrains current frameworks. For cell line profiling, SADRP employs a multi-view interactive encoder to mitigate the fragmentation inherent in independent omics processing. By leveraging contrastive multi-view learning [46,47], SADRP unifies gene expression and DNA methylation profiles to capture synergistic cross-omics complementarity. Importantly, a Save Once, Load Later (SOLL) framework is proposed to cache paired shortest paths, enabling the reuse of structural information.

## 2 Results

### 2.1 Model training and evaluation

In the mix test, we employed a balanced GDSC dataset comprising 99,594 drug-cell line pairs, covering 185 drugs and 561 cell lines. The dataset was randomly split into training, validation, and test sets with a ratio of 8:1:1. To evaluate model stability under limited data, the training set was reduced to 75%, 50%, and 25% via random sampling with a fixed seed. The same subsets were used across all models, while validation and test sets remained unchanged. IC50 distributions (S1 A) are similar across all subsets, suggesting that the sampling does not change the data distribution. All experiments were repeated five times with different random seeds, except for SADRP and DeepTTA in the data scarcity analysis, which were run ten times to enhance statistical reliability. Model performance was evaluated under several complementary settings.

Additionally, an external CCLE dataset containing 6,410 drug-cell line response pairs across 24 drugs was incorporated as an independent supplementary benchmark for evaluation.

To assess model generalization, we further conducted drug-blind, cell line-blind, and joint-blind evaluations. For the drug-blind and cell line-blind settings, drugs and cell lines were randomly partitioned into training, validation, and test sets with a ratio of 7:1:2. For the joint-blind setting, both drugs and cell lines were simultaneously partitioned with a ratio of 6:2:2. Accordingly, drugs and cell lines assigned to the test set were not observed during training, depending on the evaluation setting. Detailed statistics of the data partitions are provided in S1 Table. Furthermore, we investigated SADRP’s capability to infer unmeasured therapeutic opportunities by training the model on all available data and predicting unobserved entries in the drug-cell line response matrix.

### 2.2 Baseline methods for numerical comparison

We benchmarked SADRP against six representative baseline models. GNN- based methods including DeepCDR [33], GraphDRP [34] and MolMVC [35], Transformer-based method DeepTTA [36] and graph transformer-based methods DeepAEG [38] and MCMVDRP [39].

Model performance was quantified using Pearson’s correlation coefficient (PCC) [48], Spearman’s correlation coefficient (SCC) [49], and root mean squared error (RMSE) [50].


$$\begin{array}{c} \begin{array}{c} \text{PCC}=\frac{\sum_{i=\text{1}}^{n}(y_{i}-\bar{y})({\hat{y}}_{i}-\bar{\hat{y}})}{\sqrt{\sum_{i=\text{1}}^{n}(y_{i}-\bar{y}{)}^{\text{2}}}\sqrt{\sum_{i=\text{1}}^{n}({\hat{y}}_{i}-\bar{\hat{y}}{)}^{\text{2}}}} \end{array} \end{array}$$ (1)

$$\begin{array}{c} \begin{array}{c} \text{SCC}=\frac{\sum_{i=\text{1}}^{n}(r_{i}-\bar{r})({\hat{r}}_{i}-\bar{\hat{r}})}{\sqrt{\sum_{i=\text{1}}^{n}(r_{i}-\bar{r}{)}^{\text{2}}}\sqrt{\sum_{i=\text{1}}^{n}({\hat{r}}_{i}-\bar{\hat{r}}{)}^{\text{2}}}} \end{array} \end{array}$$ (2)

$$\begin{array}{c} \begin{array}{c} \text{RMSE}=\sqrt{\frac{\text{1}}{n}\sum_{i=\text{1}}^{n}(y_{i}-{\hat{y}}_{i}{)}^{\text{2}}} \end{array} \end{array}$$ (3)

### 2.3 Benchmarking SADRP performance

At the drug level, Belinostat and Afatinib exhibited the highest and lowest prediction accuracies, respectively (Fig 1A–1B). At the cell line level, SADRP achieved its strongest performance on BLCA and the weakest on DLBC (Fig 1C–1D), reflecting performance variability associated with inherent biological heterogeneity.

**Fig 1:**
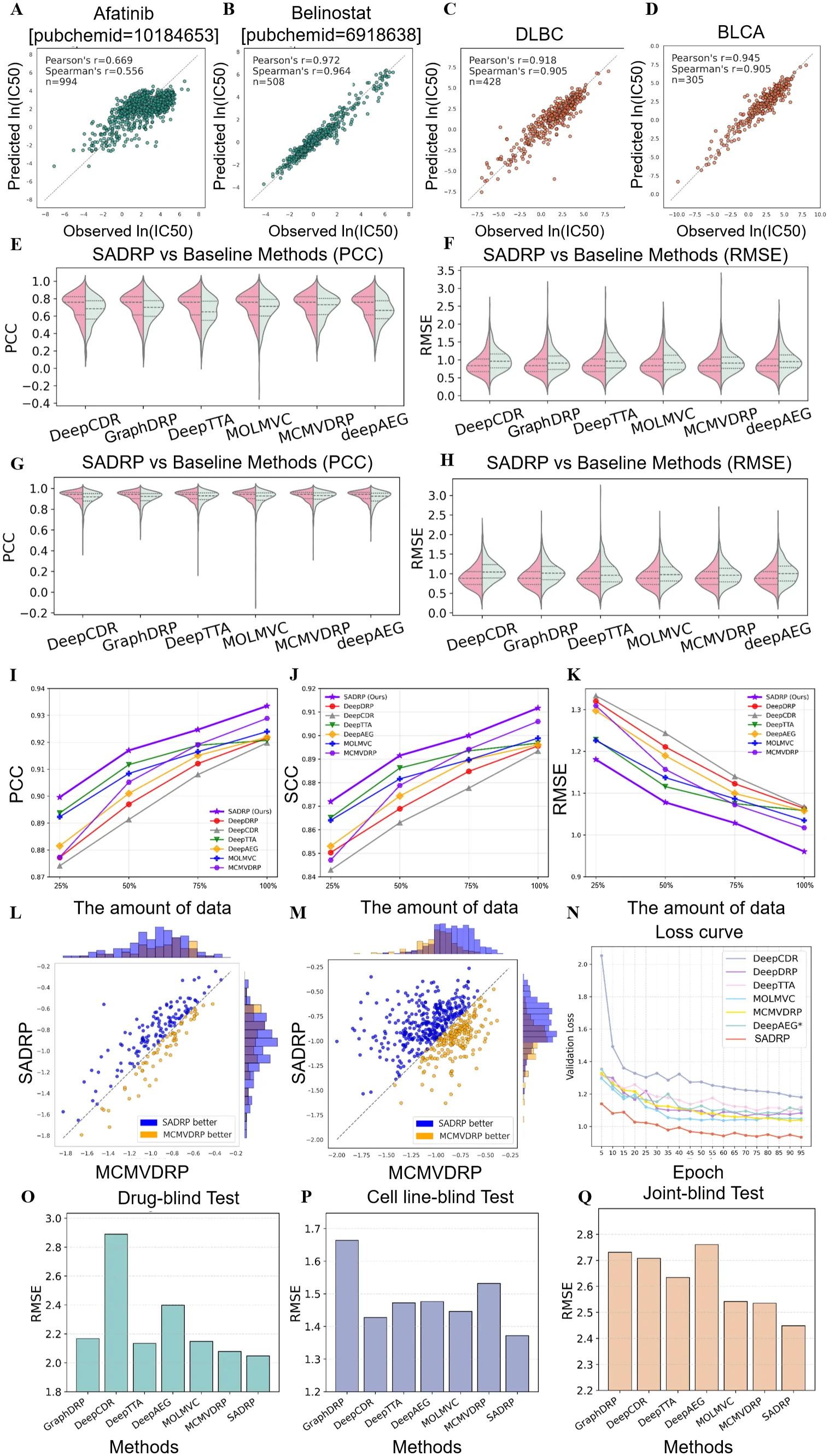
Benchmarking results of SADRP. **(A, B)** Scatter plots depicting drug-level prediction performance for the best (Belinostat) and worst (Afatinib) drugs. **(C, D)** Scatter plots showing cancer-type-level prediction performance for the best (BLCA) and worst (DLBC) cell types as predicted by SADRP. **(E, F)** Performance distributions across individual drugs for SADRP (pink) and baseline methods (green). **(G, H)** Performance distributions across individual cell lines for SADRP (pink) and baseline methods (green). Violin plots illustrate the first quartile, median, and third quartile. SADRP generally achieves higher PCC, lower RMSE, and reduced variance compared to baseline methods. **(I-K)** Model performance (PCC, SCC, and RMSE) under reduced training set sizes (75%, 50%, 25% of the full dataset), illustrating that SADRP consistently outperforms baselines. **(L, M)** RMSE per drug and per cell line. Each point represents a single drug or cell line; SADRP outperforms baseline on 123 of 185 drugs and 362 of 561 cell lines. **(N)** Validation loss across training epochs for all methods. SADRP demonstrates the fastest convergence, approaching final performance by epoch 50. Note: DeepDRP refers to the GATGCN-based version. **(O–Q)** RMSE comparison under the drug-blind, cell line-blind, and joint-blind settings, respectively. SADRP consistently achieves the lowest RMSE across all three evaluation settings, demonstrating superior generalization performance.

Performance comparisons across drug categories further supported the stability of SADRP. The model achieved higher median PCC and lower median RMSE across all drug classes, and its violin-plot distributions were more compact, reflecting improved average accuracy and reduced performance variance (Fig 1E-1F). A consistent trend was also observed in the per-cell line analysis, where SADRP exhibited higher PCC and lower RMSE distributions across individual cell lines, further demonstrating its stability and generalization ability (Fig 1G–1H).

To further evaluate the statistical significance of these performance differences, we conducted Wilcoxon signed-rank tests based on the per-drug and per-cell line PCC values. The corresponding results are summarized in S2 Table. The results indicate that the performance improvements achieved by SADRP are statistically significant across both evaluation settings (all p-values < 0.05), further supporting the robustness and reliability of the proposed framework.

Under the mixed-test setting, SADRP achieved the highest PCC and SCC, along with the lowest RMSE, among all compared methods on the GDSC and CCLE datasets (Tables 1-2). Beyond predictive accuracy, SADRP maintains a relatively compact parameter size, indicating a parameter-efficient architectural design. Notably, its performance remained superior when the training set was progressively reduced to 75%, 50%, and 25% of its original size, demonstrating stability under data-limited settings (Fig 1I-1K). Complete numerical results are provided in S3 Table.

**Table 1:** Results of the mix test on GDSC.

Method	PCC	SCC	RMSE	Parameter
GraphDRP(GAT)	0.917 ± 0.0014	0.8895 ± 0.0021	1.0826 ± 0.0077	1.6M
GraphDRP(GIN)	0.9180 ± 0.0033	0.8916 ± 0.0031	1.0919 ± 0.0163	1.44M
GraphDRP(GATGCN)	0.9216 ± 0.0017	0.8956 ± 0.0029	1.0638 ± 0.0019	4.62M
DeepCDR	0.9198 ± 0.0018	0.8934 ± 0.0011	1.0672 ± 0.0051	1.03M
DeepTTA	0.9207 ± 0.0018	0.8968 ± 0.0024	1.0585 ± 0.0158	4.05M
DeepAEG*	0.9219 ± 0.0019	0.8961 ± 0.0043	1.0578 ± 0.0111	1.65M
MOLMVC	0.9240 ± 0.0017	0.8989 ± 0.0021	1.0349 ± 0.0116	2.62M
MCMVDRP	0.9289 ± 0.0014	0.9060 ± 0.0015	1.0173 ± 0.0127	89.34M
SADRP	**0.9335 ± 0.0006**	**0.9117 ± 0.0015**	**0.9604 ± 0.005**	1.3M

**Table 2:** Results of the mix test on CCLE.

Method	PCC	SCC	RMSE	Parameter
GraphDRP(GAT)	0.8551 ± 0.0060	0.7266 ± 0.0290	1.1088 ± 0.0357	1.6M
GraphDRP(GIN)	0.8528 ± 0.0103	0.7043 ± 0.0270	1.1134 ± 0.0385	1.44M
GraphDRP(GATGCN)	0.8503 ± 0.0131	0.7219 ± 0.0299	1.1458 ± 0.0714	4.62M
DeepCDR	0.8433 ± 0.0151	0.7075 ± 0.0185	1.1515 ± 0.0274	1.03M
DeepTTA	0.8777 ± 0.0110	0.7517 ± 0.0095	1.0121 ± 0.0290	4.05M
MOLMVC	0.8841 ± 0.0110	0.7584 ± 0.0114	0.9979 ± 0.0474	2.62M
MCMVDRP	0.8534 ± 0.0149	0.7403 ± 0.0209	1.1223 ± 0.0512	89.34M
SADRP	**0.8872 ± 0.0123**	**0.7513 ± 0.0223**	**0.9658 ± 0.0338**	1.3M

To further assess the reliability of the observed performance differences under varying training data ratios, we conducted additional statistical significance analyses across repeated runs. For the comparison between SADRP and DeepTTA, the statistical tests were based on ten independent runs, whereas comparisons with all other baseline methods were based on five independent runs. The corresponding results are summarized in S4 Table. The results show that SADRP maintains statistically significant performance advantages over most baseline methods across different data availability settings, further demonstrating the stability of the proposed framework under limited training data.

SADRP’s superior generalization ability was further evidenced by head-to-head comparisons with the strongest baseline at the individual drug and cell-line levels (Fig 1L-1M). Specifically, SADRP outperformed the baseline on 362 of 561 cell lines (64.5%) and 123 of 185 drugs (66.5%).

Finally, SADRP converged faster than competing approaches; its checkpoint at epoch 50 already approached final performance (Fig 1N), underscoring its practical training efficiency.

### 2.4 Blind-test evaluation of SADRP generalization

To further assess the generalization ability of SADRP beyond the training distribution, we conducted blind tests. SADRP exhibited superior generalization in both blind-test settings (Tables 3-4), and the corresponding RMSE comparisons are presented in Fig 1O–1Q.

**Table 3:** Results of the drug blind test and cell line blind test.

Method	Drug blind test			Cell line blind test		
PCC	SCC	RMSE	PCC	SCC	RMSE
GraphDRP(GAT)	0.3767	0.3486	2.4505	0.8026	0.7592	1.6639
GraphDRP(GIN)	0.3012	0.2817	2.1815	0.7831	0.7426	1.7403
GraphDRP(GATGCN)	0.3539	0.3421	2.1672	0.8063	0.7597	1.6643
DeepCDR	0.2149	0.2193	2.8893	0.8511	0.8145	1.4273
DeepTTA	0.2872	0.3736	2.1342	0.8496	0.8094	1.4722
DeepAEG*	0.2303	0.3988	2.3988	0.8456	0.8122	1.4762
MOLMVC	0.3392	0.3859	2.148	0.8518	0.8141	1.4462
MCMVDRP	**0.4563**	0.3731	2.0782	0.8292	0.7874	1.5321
SADRP	0.4155	**0.4134**	**2.0479**	**0.8636**	**0.8302**	**1.3719**

**Table 4:** Results of the joint-blind test.

Method	PCC	SCC	RMSE
GraphDRP(GAT)	0.2563 ± 0.0798	0.2311 ± 0.0563	2.9309 ± 0.1198
GraphDRP(GIN)	0.2842 ± 0.0471	0.2094 ± 0.0324	2.7312 ± 0.0683
GraphDRP(GATGCN)	0.3091 ± 0.0486	0.2594 ± 0.0210	2.7366 ± 0.0647
DeepCDR	0.3559 ± 0.0613	0.2859 ± 0.0411	2.7080 ± 0.0929
DeepTTA	0.4174 ± 0.0365	0.3352 ± 0.0386	2.6342 ± 0.0913
DeepAEG*	0.3752 ± 0.0345	0.2921 ± 0.0349	2.7607 ± 0.0956
MOLMVC	0.4703 ± 0.0088	0.3815 ± 0.0090	2.5421 ± 0.0472
MCMVDRP	0.4570 ± 0.0082	0.2796 ± 0.0286	2.5356 ± 0.0307
SADRP	**0.4792 ± 0.0091**	**0.3956 ± 0.0145**	**2.4486 ± 0.0328**

In the cell line-blind and drug-blind tests, SADRP achieves consistently strong performance across both settings (Table 3). In the cell line-blind test, it obtains the highest PCC (0.8636), highest SCC (0.8302), and lowest RMSE (1.3719), indicating effective learning of transferable cellular representations. In the drug-blind test, all methods exhibit reduced performance due to the increased difficulty. Nevertheless, SADRP still achieves the best overall results among the compared methods.

Furthermore, under the most stringent joint-blind setting—where both drugs and cell lines in the test set are unseen during training—SADRP continues to outperform all baseline methods (Table 4). Although the overall performance decreases due to the increased difficulty, the consistent superiority of SADRP highlights its robust generalization capability across both unseen drugs and unseen cell lines.

### 2.5 Ablation study validates key architectural components

To investigate the structural characterization capabilities of SADRP, we visualized the attention maps of a representative compound, UNC1215 (PubChem ID: 57339144). The ground-truth adjacency matrix was compared with attention patterns derived from node self-attention, SDP encoding, and edge encoding (Fig 2A). Standard node self-attention produced diffuse, isotropic patterns that failed to capture the molecule’s spatial layout. In contrast, attention maps from SDP and edge encodings closely aligned with the molecular topology, indicating that these modules incorporate topological and bond-level priors.

**Fig 2:**
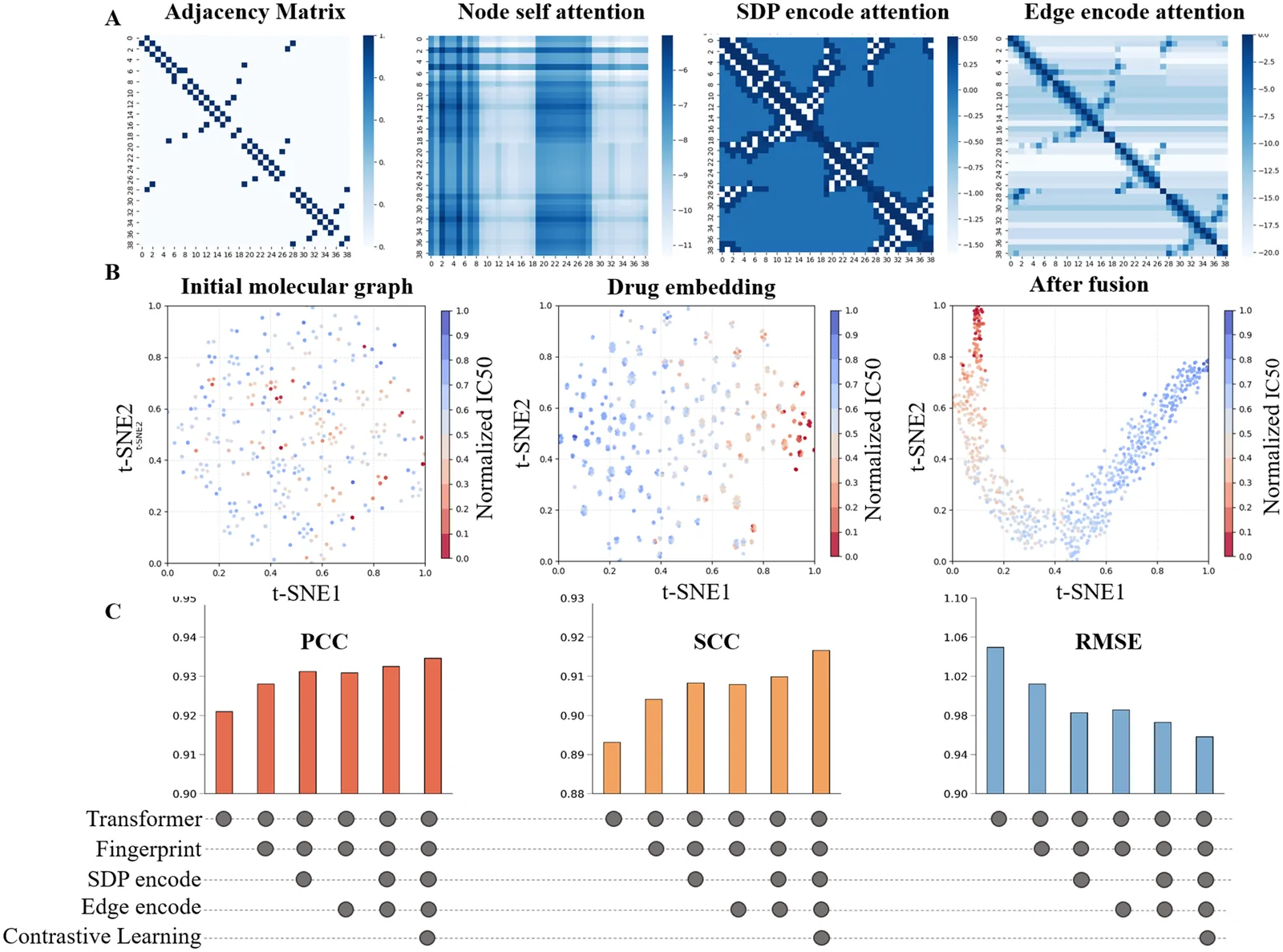
Interpretability, representation analysis, and ablation study of SADRP. **(A)** Comparison of the ground-truth adjacency matrix with attention patterns derived from node self-attention, SDP encoding, and edge encoding. The heatmaps depict how different structural components affect attention distribution over the molecular graph. **(B)** Visualization of intermediate representations at various stages of the SADRP model, showing the progression of feature extraction and multi-modal integration. **(C)** Ablation study results, evaluating the contribution of key architectural components to model predictive performance.

To illustrate progressive feature refinement, embeddings were visualized at different stages using t-SNE (Fig 2B). Raw molecular graph features showed substantial dispersion with limited structural organization. After applying the drug and cell-line encoders, more coherent clusters emerged. At the final fusion stage, representations formed compact, well-separated clusters, reflecting effective multimodal integration and enhanced feature discriminability.

We further quantified the contributions of each component via ablation experiments (Fig 2C). Incorporating molecular fingerprints consistently improved prediction accuracy, highlighting the complementary value of global chemical descriptors. SDP and edge encodings individually yielded notable performance gains; their joint integration achieved the most pronounced improvement, demonstrating a synergistic effect that captures both global spatial topology and bond-level semantics. For cell-line characterization, SADRP achieved better performance than single-modality settings (Table 5), demonstrating the importance of integrating multi-omics information for comprehensive cell-line representation learning. Furthermore, contrastive learning provided additional performance gains by improving the alignment of gene expression and methylation representations, resulting in more informative multi-omics representations for downstream drug response prediction.

**Table 5:** Results of ablation study on omics feature.

Method	PCC	SCC	RMSE
Gexpr only	0.9313 ± 0.0009	0.9101 ± 0.0019	0.9770 ± 0.0103
Methy only	0.9310 ± 0.0017	0.9087 ± 0.0015	0.9858 ± 0.0148
SADRP	**0.9335 ± 0.0006**	**0.9117 ± 0.0015**	**0.9604 ± 0.005**

To further evaluate the impact of molecular representation design, we additionally compared several alternative molecular descriptors and pretrained representations, including FP2, Atom Pair fingerprints, E3FP, and ChemBERTa embeddings (Table 6). Among all evaluated representations, Morgan fingerprints consistently achieved the best overall performance within the SADRP framework. This result suggests that Morgan fingerprints provide effective complementary substructure-level information to the Graphormer-based molecular encoder.

**Table 6:** Results of ablation study on alternative molecular descriptors and pretrained representations.

Method	PCC	SCC	RMSE
E3FP	0.9334 ± 0.0015	0.9104 ± 0.0021	0.9683 ± 0.0105
FP2	0.9315 ± 0.0014	0.9085 ± 0.0017	0.9823 ± 0.0147
ATOM PAIR	0.9320 ± 0.0014	0.9098 ± 0.0026	0.9734 ± 0.0079
ChemBERTa	0.9330 ± 0.0015	0.9108 ± 0.0022	0.9691 ± 0.0125
SADRP	**0.9335 ± 0.0006**	**0.9117 ± 0.0015**	**0.9604 ± 0.005**

### 2.6 SADRP predicts missing drug responses across drug-cell line pairs

Approximately 19.5% of the drug–cell line pairs in the original dataset (26,072 in total; Table 7) lacked IC50 annotations. We leveraged the trained SADRP model to infer these missing responses, aiming to identify potentially sensitive drug–cell line combinations. A subset of these unannotated pairs contains experimentally measured IC50 values in the GDSC2 dataset, providing an opportunity to assess prediction reliability. As summarized in Table 8, SADRP demonstrated strong concordance with the GDSC2 ground truth. For example, the predicted IC50 of Vinblastine in the breast cancer line MDA-MB-157 matched the observed value (-4.24), along with similarly accurate predictions for several other drug–cell line pairs.

**Table 7:** Comparison of the GDSC dataset before and after filtering.

	Drug	Cell line	Responses	Missing rate
GDSC (original)	238	561	107446	19.53%
GDSC (filtered)	185	561	99594	4.04%

**Table 8:** Representative cancer DRPs generated by SADRP.

Drug	Cell	Cancer	Top-5 genes	Pred.IC50	Obs.IC50
Vinblastine	MDA-MB-157	BRCA	**NFKB2** MAP2K2 CTNNB1 ZMYM3 JAK1	-4.2441	-4.2436
Docetaxel	SCC-15	HNSC	MDM4 BMP5 CNTNAP2 **EGFR** ARHGEF12	-4.0766	-4.0822
Paclitaxel	T84	COAD/ READ	**ATR** DDX5 ARID1B KDR CCR7	-3.3159	-3.3239
Paclitaxel	EFO-27	OV	BTK CDKN1A CDH11 DICER1 AR	-3.4469	-3.4275
Erlotinib	SU-DHL-10	DLBC	AOBEC3B CBFB **STAT3** MDM2 ERCC2	1.5449	1.6258

Beyond individual predictions, SADRP revealed broader patterns of drug activity. Fig 3A depicts predicted IC50 distributions for unannotated compounds. Several well-established anticancer agents, including Bortezomib, Docetaxel, and Daporinad, consistently exhibited low predicted IC50 across multiple cell lines, indicating successful recovery of compounds with known cytotoxic profiles. To further interpret these observations from a biological perspective, we performed GO and KEGG pathway enrichment analyses on the target genes of drugs exhibiting low IC50 values using the DAVID platform.

**Fig 3:**
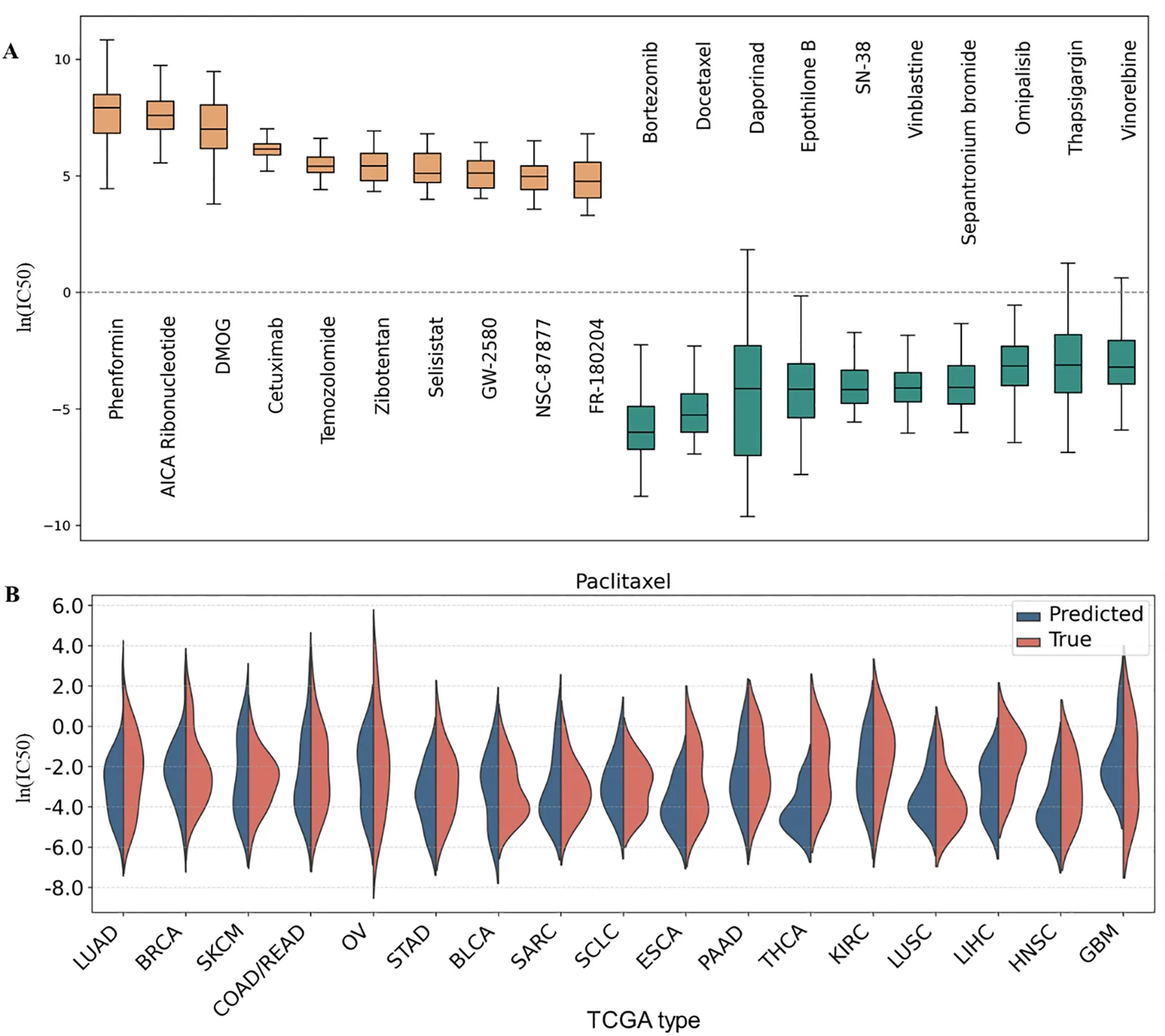
SADRP predicts missing drug-cell line responses. **(A)** Bar plot showing the mean predicted ln(IC50) values for ten most and least sensitive drugs among missing drug-cell line pairs. **(B)** Comparison of true and predicted ln(IC50) values for Paclitaxel across multiple TCGA cancer types, illustrating cancer-type-specific drug responses captured by the model.

We further examined cancer-type-specific response patterns. Using Paclitaxel as an example, Fig 3B compares predicted and observed IC50 values across TCGA cancer types. The close agreement across diverse tumor lineages indicates that SADRP captures major trends in the drug response landscape and generalizes to heterogeneous cancer contexts. Collectively, these analyses demonstrate the model’s ability to fill large-scale data gaps and generate biologically meaningful predictions that may guide downstream experimental validation.

### 2.7 SADRP identifies JW-7-52-1 as a potential anticancer candidate

We next explored whether SADRP could identify previously under-characterized compounds with potential anticancer activity. We focused on drug–cell line pairs that remained unmeasured in both GDSC1 and the subsequently released GDSC2 dataset, thereby prioritizing responses that have not yet been experimentally characterized. Among drugs with a sufficient number of such unmeasured response pairs, JW-7-52-1 (PubChem ID: 49836027) was identified as the top-ranked compound based on predicted IC50 values. Fig 4A shows the predicted IC50 landscape of JW-7-52-1, a preclinical small molecule with limited pharmacological annotation. The model predicts high sensitivity across multiple cancer types, with ln(IC50) values largely below -2, and particularly strong responses in diffuse large B-cell lymphoma (DLBC), medulloblastoma (MB), and esophageal carcinoma (ESCA). These predictions suggest that JW-7-52-1 may possess potent cytotoxic activity, indicating potential for further experimental validation.

**Fig 4:**
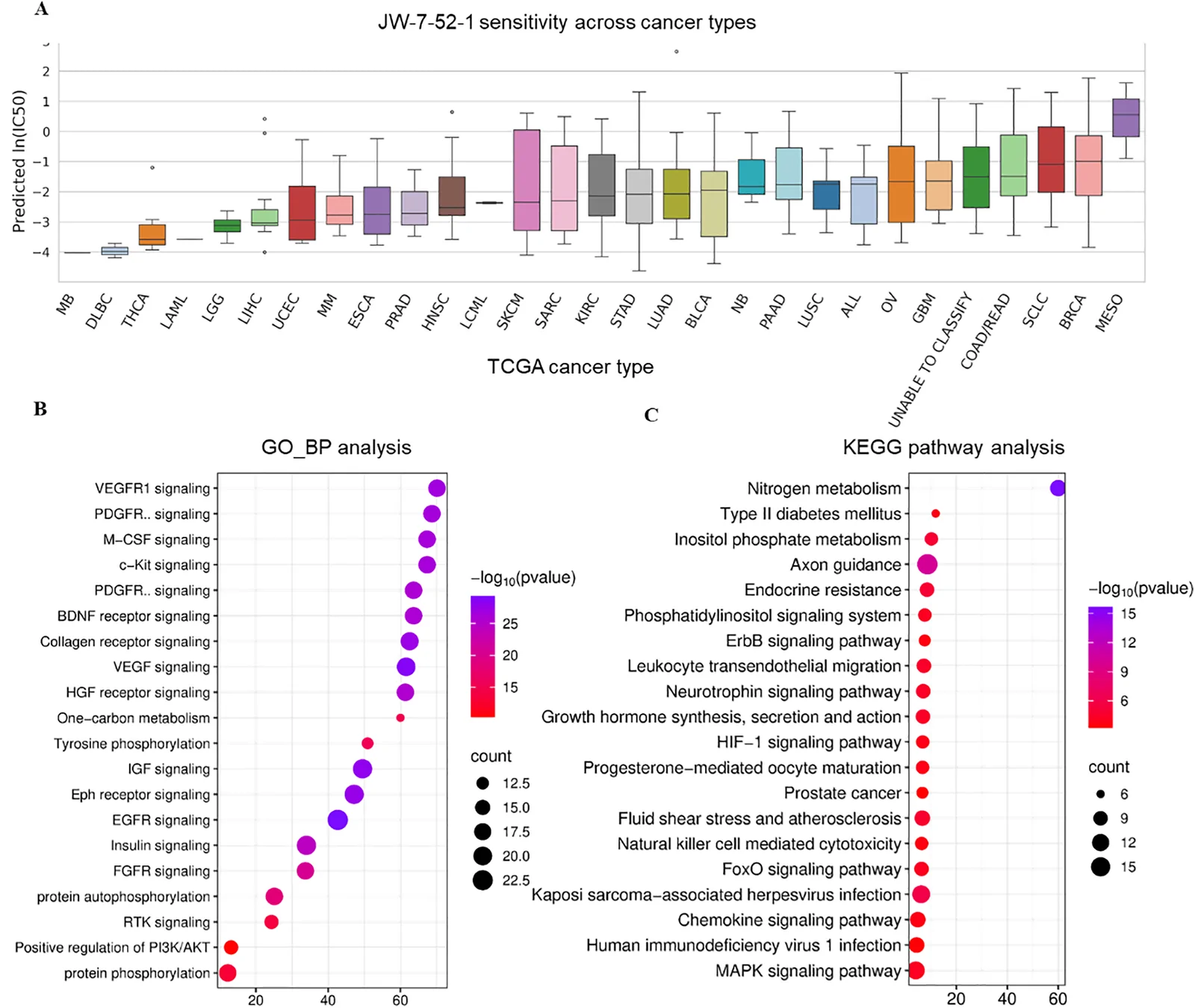
Predicted drug response and functional analysis of JW-7-52-1. **(A)** Bar plot showing the mean predicted ln(IC50) of JW-7-52-1 across cell lines from various TCGA cancer types. **(B)** GO Biological Process enrichment analysis of JW-7-52-1 target genes, depicting potential mechanisms of predicted anticancer activity. **(C)** KEGG pathway enrichment analyses of JW-7-52-1 target genes, depicting potential mechanisms of predicted anticancer activity.

To investigate possible mechanisms of action, we performed an integrative functional analysis based on putative target genes of selected compounds, with the relevant target gene data sourced from databases including CTD [51], SwissTargetPrediction [52], and PharmMapper [53]. GO Biological Process enrichment [54] of JW-7-52-1 (Fig 4B-4C) revealed strong association with receptor tyrosine kinase (RTK)-related signaling, including EGFR, VEGFR, IGF, PDGF, and FGFR pathways—key regulators of oncogenic proliferation, survival, and angiogenesis. These pathways converge on PI3K/AKT signaling and protein phosphorylation processes, implicating JW-7-52-1 as a putative multi-target kinase modulator.

Such RTK-linked signaling profiles align with the high predicted sensitivity in DLBC, MB, and ESCA, where dysregulated RTK activity is well documented. In DLBC, EGFR is frequently upregulated and activates PI3K/AKT signaling, while PDGFD/PDGFR axis promotes ibrutinib resistance via EGFR crosstalk. In medulloblastoma, multiple RTKs (including PDGFR, IGF-1R, and EGFR) drive proliferation, survival, and migration through Akt pathway activation [55–57]. In esophageal cancer (ESCC/EAC), EGFR is overexpressed or amplified in a large proportion of cases (often >50–80%), frequently co-occurring with FGFR alterations, correlating with tumor progression and poor prognosis; EGFR inhibitors such as icotinib or gefitinib have shown clinical activity in EGFR-overexpressing/amplified patients, improving response rates especially when combined with radiotherapy. These findings substantiate the translational relevance of JW-7-52-1 as a multi-RTK modulator for these RTK-driven malignancies.

### 2.8 SADRP identifies cancer-related genes

To investigate the biological signals learned by SADRP, we examined whether the model could recover cancer-relevant genes associated with specific drug–cell line responses. Unlike prior approaches such as DeepCDR [33], which mainly rely on gradient-based attribution, we employed SHAP [58] to quantify the contribution of individual gene expression features. We focused on 697 genes curated in the COSMIC Cancer Gene Census [59], representing known drivers and regulators of tumorigenesis.

For each drug-cell line pair, we fixed a given drug and selected 64 cell lines treated with the same agent as the background reference. SHAP was then applied to generate 3,442 perturbation evaluations across the 697-dimensional gene expression space. Genes were ranked by absolute SHAP values, and the top five were retained as key contributors for each prediction.

Strikingly, many of the genes identified by SADRP correspond to well-established regulators of drug response or cancer progression reported in the literature (Table 8). For example, in the breast cancer cell line MDA-MB-157, NFKB2 emerged as a top contributor to Vinblastine response. Vinblastine, a microtubule-destabilizing agent, is known to activate NF-κB signaling by promoting IκBα degradation and IKK activation, thereby inducing apoptosis in malignant cells [60]. Similarly, in the colorectal cancer cell line T84, ATR was highlighted as a major determinant of Paclitaxel response. ATR plays a critical role in maintaining chromosomal stability and centromere integrity via R-loop regulation, processes directly linked to taxane-induced mitotic catastrophe [61,62]. The concordance between the model-identified genes and previously reported biological mechanisms supports the reliability of SADRP in capturing meaningful gene–drug associations.

These findings demonstrate that SADRP not only achieves accurate predictions but also provides biologically meaningful insights, highlighting its potential utility for guiding biomarker discovery and informing personalized therapeutic strategies.

## 3 Discussion

In this study, we developed SADRP, a structurally aware multi-modal deep learning framework for anticancer drug response prediction. SADRP integrates enhanced molecular graph representations of drugs with multi-omics profiles of cancer cell lines. By integrating a Graphormer-based molecular encoder, a Morgan fingerprints encoder and a multi-view interactive omics encoder for gene expression and DNA methylation data, the model captures both complex structural features of compounds and cross-omics complementarity in cell lines. We comprehensively evaluated SADRP using multiple data splitting strategies on the GDSC dataset, including mixed, drug-blind, cell line-blind, and joint-blind tests. In addition, we evaluated SADRP on an independent benchmark constructed from the CCLE dataset by performing a mixed test. The results demonstrate that SADRP consistently achieves superior performance compared with state-of-the-art methods across Pearson correlation coefficient, Spearman correlation coefficient, and RMSE metrics, while maintaining parameter efficiency and training stability under reduced data conditions.

The innovative aspects of SADRP lie in its structurally aware drug encoder and interactive multi-omics encoder. For drug representation, we enhanced the Transformer-based architecture with SDP encoding to capture topological distances between atoms and edge encoding to incorporate bond-type chemical semantics. These additions address the limitations of standard graph neural networks and sequence-based transformers in modeling global molecular contexts and edge-level information. For cell line representation, we designed a multi-view interactive encoder that uses contrastive learning to unify gene expression and DNA methylation profiles, enabling the model to learn synergistic information across omics layers rather than processing them independently. The SOLL (Save Once, Load Later) framework further improves computational efficiency by caching shortest-path information, allowing practical training on large numbers of drug-cell line pairs.

A key strength of SADRP is its ability to provide mechanistic insights through attention patterns and feature attribution. Attention map analysis on representative compounds showed that SDP and edge encodings effectively capture molecular topology and bond semantics, producing attention patterns that align closely with actual chemical structures. Embedding visualizations at different stages confirmed progressive refinement of features and effective multimodal integration. In downstream analyses, SADRP successfully inferred missing drug-cell line responses with high concordance to independent GDSC2 measurements. It also identified JW-7-52-1 as a promising candidate with predicted broad sensitivity, particularly in DLBC, medulloblastoma, and esophageal carcinoma. Functional enrichment analysis linked this compound to receptor tyrosine kinase signaling pathways, including EGFR, PI3K/AKT, and related processes, consistent with known biology in these cancer types. Furthermore, SHAP-based attribution on COSMIC cancer genes recovered biologically meaningful associations, such as NFKB2 in Vinblastine response and ATR in Paclitaxel response, aligning with established mechanisms in the literature.

Although SADRP demonstrates strong predictive performance and interpretability, several limitations should be acknowledged. First, although we integrated gene expression and DNA methylation, other important omics layers such as proteomics, metabolomics, and mutation data were not included; incorporating additional data types could provide a more complete view of cellular states. Second, while SADRP shows good generalization on known compounds, its performance on entirely novel drug candidates remains limited. Third, although the model offers reasonable interpretability through attention and SHAP analyses, explaining complex interactions in high-dimensional multi-modal settings can still be challenging. In future work, we plan to integrate additional omics layers, explore more advanced pre-trained molecular models to improve generalization to new chemical entities, investigate alternative graph transformer architectures, and enhance the interpretability modules. Expanding the framework with clinical data will further support its translation toward personalized cancer therapy.

## 4 Materials and methods

### 4.1 Datasets and preprocessing

Data used in this study were obtained from three public resources: the Genomics of Drug Sensitivity in Cancer (GDSC) [63], the Cancer Cell Line Encyclopedia (CCLE) [64], and PubChem [65]. Log-transformed IC50 values, representing drug sensitivity for drug-cell line pairs, were obtained from GDSC. Multi-omics profiles, including gene expression and DNA methylation data, were retrieved from CCLE via the DepMap portal [66], which provides comprehensive molecular characterizations of cancer cell lines.

We first aligned the drug response data with the corresponding multi-omics profiles of cell lines. To reduce missing values, we retained only those drugs with IC50 measurements available in more than half of the cell lines. This filtering step lowered the proportion of missing values from 19.53% to 4.04% (Table 7). To evaluate the potential impact of this filtering strategy, we further compared the distributions of IC50 values and molecular sizes before and after filtering. As shown in S1B–S1C Fig, the distributions remained visually similar. We further performed two-sample Kolmogorov–Smirnov (KS) tests. The molecular size distribution showed no statistically significant difference after filtering. For the IC50 distribution, the corresponding KS statistic was only 0.0103, indicating that the distributional change was minimal.

For gene expression data, features were restricted to COSMIC genes following the preprocessing protocol of DeepCDR. Prior to model training, both gene expression and DNA methylation features were standardized using z-score normalization, resulting in zero mean and unit variance for each feature. No additional transformation, feature filtering or batch effect correction was applied.

In addition, we incorporated drug-response profiles from the CCLE pharmacological screening dataset. After aligning these responses with our curated cell line collection, we obtained 24 drugs and 6,410 drug-cell line pairs.

The CCLE dataset was treated as an independent benchmark, where a random split at the drug–cell line pair level was performed, following the same protocol as the mixed test on GDSC.

### 4.2 Model architecture

In this study, we analyze molecular graph structures, molecular fingerprints, and multi-omics data at the atom level, substructure level, and cell line level, respectively, to uncover the associations between drug compounds and cell line responses. SADRP consists of five key components (Fig 5A–E): (1) a Graphormer-based molecular encoder, (2) a Morgan fingerprint encoder, (3) a multi-view interactive omics encoder, (4) a prediction module, and (5) a GO/KEGG enrichment analysis module for biological interpretation.

#### 4.2.1 Graphormer-based molecular encoder.

##### Drug Molecular Graph Construction.

We initially construct a drug molecular graph, which is composed of node attributes, edge connections, and edge features, forming the basis for accurate molecular representation. Using RDKit [67], SMILES strings are converted into molecular graphs G=(V,E). The graph is characterized by three components: (i) a node feature matrix $𝐗\in R^{N_{node}\times f_{n}}$, where $N_{node}$ is the number of atoms and each atom is encoded as a 21-dimensional feature vector $f_{n}=\text{21}$ (S5 Table); (ii) an adjacency matrix $𝐀\in R^{N_{node}\times N_{node}}$, which encodes atomic connectivity; (iii) an edge feature matrix $𝐄\in R^{N_{edge}\times f_{e}}$, where $N_{edge}$ is the number of chemical bonds and each bond is encoded as an 11-dimensional feature vector $f_{e}=\text{11}$ (S6 Table). Since node features and edge features are primarily binary or low-range discrete variables, no explicit normalization is applied.

The node feature matrix 𝐗 is processed by a two-layer GCN to produce a transformed 128-dimensional representation matrix ${𝐗}^{\prime }$. Meanwhile, edge feature matrix 𝐄 is encoded by an MLP to obtain 64-dimensional representation matrix ${𝐄}^{\prime }$:


$$\begin{array}{c} \begin{array}{c} {𝐗}^{\prime }={GCN}_{\text{2}}\left(PReLU\left({GCN}_{\text{1}}\left(𝐗,𝐀\right)),\;𝐀\right)\right)\;\;\;\;,\;{𝐗}^{\prime }\in R^{N_{node}\times \text{128}} \end{array} \end{array}$$ (4)

$$\begin{array}{c} \begin{array}{c} {𝐄}^{\prime }=MLP(𝐄)\;,\;{𝐄}^{\prime }\in R^{N_{edge}\times \text{6}\text{4}} \end{array} \end{array}$$ (5)

where PReLU is a ReLU variant with a learnable slope for negative inputs, enhancing gradient flow and model flexibility.

##### Graphormer-based molecular encoder with SDP and edge encoding.

Conventional graph transformers inherently treat molecular graphs as fully connected systems, assigning equivalent significance to all atom pairs regardless of their topological distance [45]. However, the biological activity of a drug is often dictated by pharmacophores, which consist of specific arrangements of atoms in close proximity [68]. To capture this, we utilize Shortest Distance Path (SDP) encoding to reflect the spatial decay of atomic influences, prioritizing interactions within local structural motifs. Furthermore, the chemical identity of a molecule is defined not only by its atoms but also by its bond types and connectivity, which critically influence molecular conformation and target binding behavior. Edge encoding is introduced to incorporate this bond-level information.

In SADRP, these structural descriptors are supplemented into the node self-attention mechanism via learnable coefficients (α), which adaptively modulate the influence of topological and bond-level information during training. By jointly capturing positional and bond-level cues, these enhancements enable the model to generate more expressive and chemically meaningful molecular representations for drug response prediction.

**Node self-attention Mechanism.** Let ${𝐗}^{\prime }=[x_{\text{1}},x_{\text{2}},\ldots ,\;x_{n}]$ denote the input to the self-attention module where $x_{i},x_{j}\in R^{\text{1}\times \text{128}}$ represent the feature of node i,j. Given the node representations, the self-attention score ${𝐀}_{ij}$ between node i and node j is computed as:


$$\begin{array}{c} \begin{array}{c} {𝐀}_{ij}=\frac{\left(x_{i}{𝐖}_{Q}\right){\left(x_{j}W_{K}\right)}^{T}}{\sqrt{d}} \end{array} \end{array}$$ (6)

where ${𝐖}_{𝐐}\in R^{\text{128}\times \text{128}}\text{ denotes query metrics}$, ${𝐖}_{𝐊}\in R^{\text{128}\times \text{128}}$ denotes key matrices and d denotes scaling factor.

**SDP Encoding in Attention.** In molecular graphs, the number of bonds between two atoms reflects how strongly they can influence each other. Chemical effects such as electronic interaction, steric influence, and substituent impact typically decay as the topological distance increases, making distance encoding crucial for molecular feature extraction.

To encode the structural distance between node pairs, we introduce a set of learnable positional embedding vectors $b\in R^{D}$. The shortest distance path (SDP) encoding between node i and node j is defined as [45]:


$$\begin{array}{c} \begin{array}{c} {𝐒𝐃𝐏}_{i,j}=b_{\tau \left(d_{i,j}\right)},\tau \left(d_{i,j}\right)=\text{min}\left(d_{i,j},D_{\text{max}}\right) \end{array} \end{array}$$ (7)

where $d_{i,j}$ denotes the shortest-path distance between nodes iand j, and τ(·) is a truncation function that caps the distance at a predefined maximum $D_{\text{max}}$.

**Edge Encoding in Attention.** In molecular graphs, chemical bonds differ in length, strength, and electron distribution (e.g., single, double, or aromatic bonds), which influence molecular stability, reactivity, and conjugation. However, conventional GNNs cannot explicitly encode such bond-level distinctions. Edge encoding addresses this limitation and enhances the representational capacity of molecular graphs.

Specifically, given an ordered node pair $\left(v_{i},v_{j}\right)$, we identify the shortest path ${SP}_{ij}=\left(e_{\text{1}},e_{\text{2}},\ldots ,e_{n}\right)\in {𝐄}^{\prime }$ that connects them in the molecular graph. Along this path, we compute the average dot products between each edge feature and a learnable edge embedding vector. The resulting edge encoding between node i and node j is defined as [45]:


$$\begin{array}{c} \begin{array}{c} {𝐄}_{i,j}=\;\frac{\text{1}}{N}\sum_{n=\text{1}}^{N}x_{e_{n}}{\left(\omega_{n}^{E}\right)}^{T} \end{array} \end{array}$$ (8)

where $x_{e_{n}}$ denotes the feature vector of the n-th edge $e_{n}$ along the path ${SP}_{ij}$, $\omega_{n}^{E}\in R^{d_{E}}$ is its corresponding learnable embedding vector, and $d_{E}$ is the dimensionality of edge feature space.

**Attention Sum-up.** To integrate multiple sources of structural information, the final attention matrix 𝐀𝐭𝐭 is computed by combining the Node self-attention with the SDP encoding and edge encoding:


$$\begin{array}{c} \begin{array}{c} 𝐀𝐭𝐭=𝐀+\alpha \cdot 𝐒𝐃𝐏+(\text{1}-\alpha )\cdot 𝐄 \end{array} \end{array}$$ (9)

where 𝐀 denotes node self-attention matrix, 𝐒𝐃𝐏 represents the shortest-path distance encoding, and 𝐄 denotes the edge feature encoding matrix. The scalar parameter α∈[0,1], initialized to 0.5, is learnable and used to balance the contributions between SDP encoding and edge encoding.

**Embedding Generation.** Given the fused attention matrix $𝐀𝐭𝐭\in R^{N\times N}$, the node embeddings ${𝐗}^{*}$ are generated by the multi-head attention mechanism (MHA) as:


$$\begin{array}{c} \begin{array}{c} {𝐗}^{*}=MHA\left(Softmax\left(𝐀𝐭𝐭\right)\;𝐕\right),\;𝐕=𝐗{𝐖}_{𝐯} \end{array} \end{array}$$ (10)

where ${𝐖}_{𝐯}\in R^{\text{128}\times \text{128}}$ denotes the value projection matrix, $𝐗\in R^{N\times \text{128 }}$ s the input node-feature matrix, and $𝐕=𝐗{𝐖}_{𝐯}\in R^{N\times \text{128}}\;$ denotes the projected value matrix.

After feature extraction by Graphormer-based encoder, the node embeddings ${𝐗}^{*}$ are passed through global mean pooling to derive the final graph-level representation. Among several pooling strategies (mean, max, mean-max, and gated pooling), mean pooling achieved the best performance, likely because the self-attention mechanism already captures rich inter-atomic dependencies, making simple aggregation sufficient and stable.


$$\begin{array}{c} \begin{array}{c} {drug\;embedding}_{\text{1}}=\;\frac{\text{1}}{n}\;\sum_{i=\text{1}}^{n}{𝐗}^{*} \end{array} \end{array}$$ (11)

While the proposed model effectively integrates heterogeneous information across multiple levels, computational complexity remains a key challenge during molecular graph processing. To alleviate this, we developed the SOLL (Save Once, Load Later) framework, which optimizes shortest-path computations and accelerates model training (Section 2.3).

#### 4.2.2 Morgan fingerprints encoder.

To complement the graph-based features, each drug molecule is also encoded using a 512-bit Morgan fingerprint, a fixed-length binary vector in which each bit indicates the presence of a particular circular substructure. This representation captures local chemical environments, including functional groups, ring systems, and characteristic bonding patterns. A two-layer MLP transforms these sparse, high-dimensional vectors into dense embeddings that preserve essential substructural information. The resulting representations provide a complementary view to the graph-based encodings, supporting the model’s multi-modal fusion for DRP.


$$\begin{array}{c} \begin{array}{c} {drug\;embedding}_{\text{2}}=ReLU\left(\omega_{2}\left(ReLU\left(\omega_{1}fp+\;b_{\text{1}}\right)\right)\;+\;b_{\text{2}}\right) \end{array} \end{array}$$ (12)

where fp denotes Morgan fingerprint.

#### 4.2.3 Multi-view interactive encoder.

The **Multi-view interactive encoder** employs a contrastive learning strategy to capture synergistic correlations between gene expression (*G*) and DNA methylation (M) profiles. A contrastive learning framework jointly encodes these profiles into a shared latent space, generating cell line representations that can be integrated with drug embeddings. The prediction module then estimates IC50 values using linear layers based on the combined latent representations.

In a mini-batch of size B, omics features represent as (G, M), where $G\in R^{B\times d_{g}}$, and $M\in R^{B\times d_{m}}$, are processed through separate encoders. Each encoder is implemented as a two-layer MLP, consisting of two fully connected layers with dimensions $d_{in}\to \text{2}\text{5}\text{6}\to \text{1}\text{2}\text{8}$, where 256 is the hidden dimension and 128 is the output embedding dimension. This yields projected embeddings $Z_{g}=f_{g}(G_{i})$ and $Z_{m}=f_{m}(M_{i})$ for gene expression and methylation, where $Z_{g},\;Z_{m}\in R^{B\times d^{\prime }}$.

Multi-omics data provide complementary views of cellular states beyond any single modality. Despite capturing distinct modality-specific features, omics profiles from the same cell line are expected to share high-level semantic consistency, while remaining separable across different cell lines. To encourage such cross-omics semantic consistency while preserving modality-specific characteristics, we adopt an InfoNCE contrastive loss [69]:


$$\begin{array}{c} \begin{array}{c} Loss=\;-\frac{\text{1}}{B}\sum_{i=\text{1}}^{B}\text{log}\frac{\text{exp}\left(\frac{{\left(z_{g}^{i}\right)}^{⊤}z_{m}^{i}}{\tau }\right)}{\sum_{j=\text{1}}^{B}\text{exp}\left(\frac{{\left(z_{g}^{i}\right)}^{⊤}z_{m}^{j}}{\tau }\right)} \end{array} \end{array}$$ (13)

where $z_{g}^{i}$ and $z_{m}^{i}$ denote the embeddings of gene expression and methylation for the i-th sample, respectively. The similarity is computed as the dot product ${\left(z_{g}^{i}\right)}^{⊤}z_{m}^{j}$ (where jranges over all samples in the mini-batch). The temperature parameter τ is set to 0.1.

During contrastive pretraining, the InfoNCE loss is computed within each mini-batch. For a batch of size B, each paired sample $\left(z_{g}^{i},z_{m}^{i}\right)$ forms a positive pair, while all other combinations $(z_{g}^{i},z_{m}^{j}),i\neq j$, are treated as negative pairs. The batch size is set to 32. This loss encourages paired omics representations to cluster closely while pushing apart mismatched pairs, reflecting the assumption that different omics profiles derived from the same biological entity should share common semantics in the latent space. The pretrained omics encoder is subsequently integrated into SADRP for downstream drug response prediction, where the model is optimized using the MSE loss.

#### 4.2.4 Predictor.

In the predictor module, drug embeddings derived from atom-level and substructure-level representations, together with cell line embeddings, are first concatenated and then passed through a 3-layer MLP for IC50 prediction. Specifically, the concatenated representation is processed by two hidden fully connected layers, each followed by a ReLU activation function and a dropout layer, to enhance nonlinear representation learning and reduce overfitting. The final fully connected layer outputs the predicted IC50 value.

**Fig 5:**
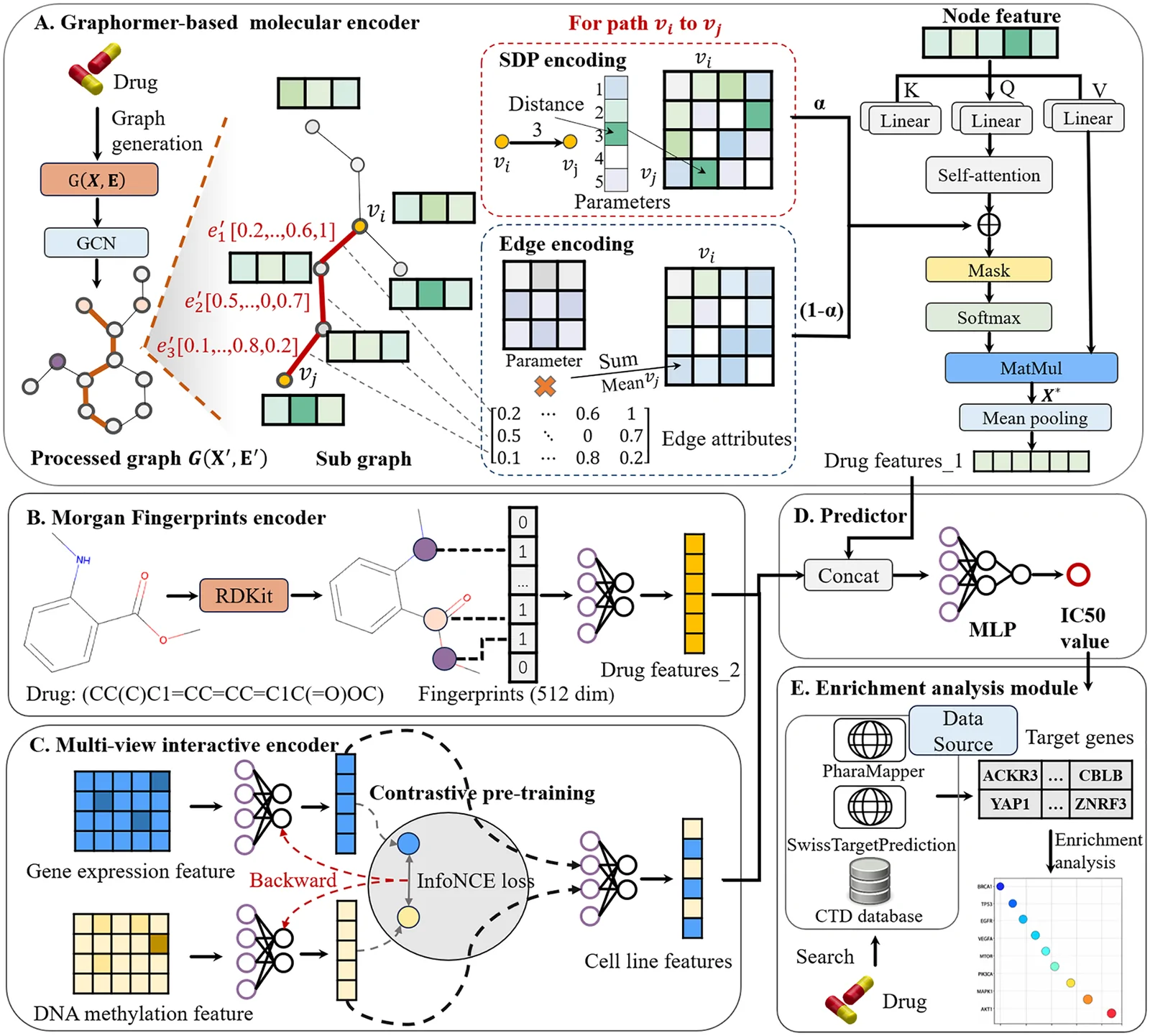
Overview of the SADRP architecture. SADRP comprises five key components: **(A)** Graphormer-based molecular encoder capturing both topological and chemical contexts from molecular graphs through atom-level and bond-level representations. **(B)** Morgan Fingerprints encoder that extracts substructural drug features from 512-bit Morgan fingerprints. **(C)** Multi-view interactive encoder that integrates gene expression and DNA methylation features from cell lines. **(D)** Predictor module that concatenates multi-modal features, including atom-level and substructure-level drug embeddings as well as cell line representations, and feeds them into a multi-layer perceptron (MLP) to predict the final IC50 values. **(E)** Enrichment analysis module that identifies biological functions and signaling pathways associated with drug-target genes, providing mechanistic insights into drug actions.

### 4.3 SOLL framework for efficient training

To improve the computational efficiency of SDP and edge encoding in SADRP, we propose the SOLL (Save Once, Load Later) framework, which combines shortest-path precomputation, and tensorized implementations to improve GPU efficiency. The framework (Fig 6) operates in two stages. During the first iteration, the Floyd-Warshall algorithm [70] is executed to obtain shortest paths between all node pairs in the graph and cache the results in a Path Triplet data structure. In subsequent iterations, cached triplets are retrieved via graph hashing, thereby eliminating redundant computation. The key components of SOLL are as follows:

**Fig 6:**
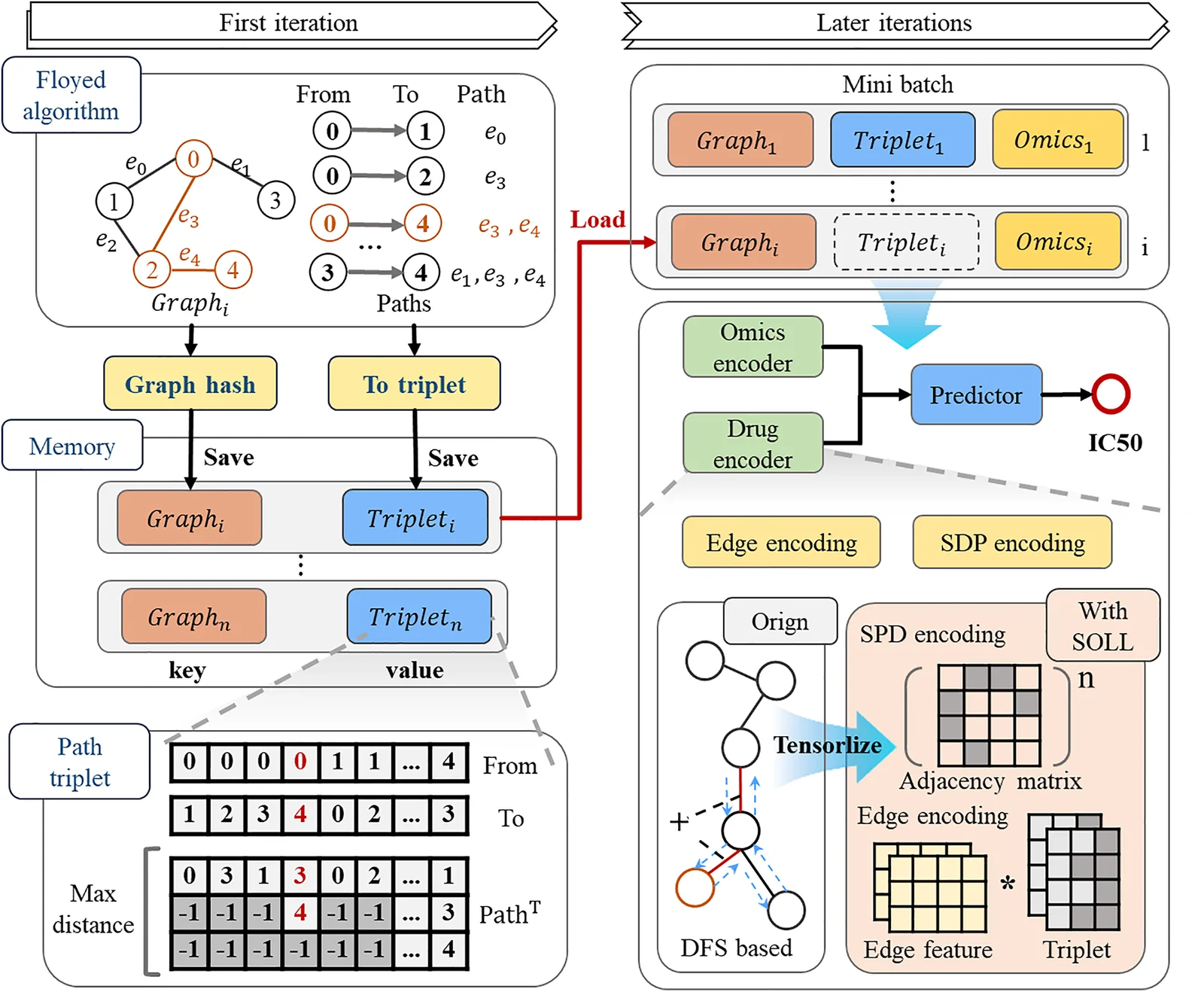
Overview of the SOLL framework and the tensorized Path Triplet representation. SOLL performs all-pair shortest-path computation in the first iteration using the Floyd–Warshall algorithm. The resulting shortest-path information is cached in memory via a key–value design that maps each molecular graph’s hash to its Path triplet representation. In later iterations, graphs are retrieved via hashing to access their stored triplets directly, enabling tensor-based access to path information without recomputing shortest paths. This design replaces recursive or DFS-style procedures with tensor operations, substantially improving GPU utilization, reducing training cost.

(1) **Path triplet data structure.** The Triplet structure stores all pairwise shortest paths in a graph using only three tensors. The From and To tensors record the source and target nodes of each path, while the Path tensor contains the sequence of edges along the shortest path between each node pair. Path sequences are truncated to a predefined maximum length and padded with −1 when shorter than this limit.
(2) **Caching strategy in SOLL.** In SOLL, a key–value map is employed in which the hashed graph as the key and its corresponding Triplet as the value. This design enables O(1)-time retrieval of precomputed shortest-path information in subsequent iterations, eliminating redundant graph-structural computations.
(3) **Tensorization of SDP and edge encodings.** With the Triplet structure in place, the originally DFS-based procedures for SDP encoding and edge encoding are reformulated as tensorized operations. SDP encoding is transformed into adjacency-matrix multiplication, while edge encoding is rewritten as matrix multiplications between edge features and the triplet tensors. Detailed implementations are provided in our GitHub repository, including the tensorized realization of SDP and edge encodings.

To facilitate reproducibility and reuse, we additionally provide a standalone implementation of SOLL together with an executable demo in our GitHub repository, allowing the framework to be readily integrated into other graph neural network models. Quantitative comparisons between implementations with and without SOLL are summarized in S7 Table. The results show that SOLL substantially reduces GPU memory consumption (from 15.86 GB to 6.60 GB) while increasing the feasible batch size from 16 to 128. In addition, the per-epoch training time is reduced from 81.0 min to 6.31 min, accompanied by a substantial improvement in GPU utilization. These results demonstrate that the proposed caching and tensorization strategies improve training efficiency and hardware utilization in practice.

## Supporting information

S1 Fig Label and molecular size distributions before and after filtering, and under different data ratios. (TIF)

S1 Table Statistics of the drug-blind, cell line-blind, and joint-blind data splits. (XLSX)

S2 Table P-values from the Wilcoxon signed-rank test for per-drug and per-cell line PCC improvements of SADRP over baseline methods. (XLSX)

S3 Table The mixed test results on different training data ratio. (XLSX)

S4 Table P-values of statistical significance tests under different training data ratios. (XLSX)

S5 Table Node features to construct graph. (XLSX)

S6 Table Edge features to construct graph. (XLSX)

S7 Table Computational efficiency comparison of SOLL and non-SOLL implementations. (XLSX)

## References

[1] Wan Z, Sun X, Li Y, Chu T, Hao X, Cao Y, et al. Applications of artificial intelligence in drug repurposing. Advanced Science. 2025;12(14):2411325. 10.1002/advs.202411325 PMC11984889 40047357

[2] Le J, Dian Y, Zhao D, Guo Z, Luo Z, Chen X, et al. Single-cell multi-omics in cancer immunotherapy: from tumor heterogeneity to personalized precision treatment. Mol Cancer. 2025;24(1):221. doi: 10.1186/s12943-025-02426-3 40855431 PMC12376342

[3] Wu Y, Chen M, Qin Y. Anticancer drug response prediction integrating multi-omics pathway-based difference features and multiple deep learning techniques. PLoS Comput Biol. 2025;21(3):e1012905. doi: 10.1371/journal.pcbi.1012905 40163555 PMC11978092

[4] Mao Y, Shangguan D, Huang Q, Xiao L, Cao D, Zhou H, et al. Emerging artificial intelligence-driven precision therapies in tumor drug resistance: recent advances, opportunities, and challenges. Mol Cancer. 2025;24(1):123. doi: 10.1186/s12943-025-02321-x 40269930 PMC12016295

[5] Qiao J, Jin J, Wang D, Teng S, Zhang J, Yang X, et al. A self-conformation-aware pre-training framework for molecular property prediction with substructure interpretability. Nat Commun. 2025;16(1):4382. doi: 10.1038/s41467-025-59634-0 40355450 PMC12069555

[6] Chen S, Yan K, Li X, Liu B. Protein Language Pragmatic Analysis and Progressive Transfer Learning for Profiling Peptide-Protein Interactions. IEEE Trans Neural Netw Learn Syst. 2025;36(8):15385–99. doi: 10.1109/TNNLS.2025.3540291 40100664

[7] Yan K, Xiang K, Chen Z, Chen S, Lu S, Liu B, et al. TPpred-CMvL: prediction of multi-functional therapeutic peptide using contrast multi-view learning. BMC Biol. 2025;23(1):363. doi: 10.1186/s12915-025-02466-7 41462221 PMC12752346

[8] Qi R, Ma A, Ma Q, Zou Q. Clustering and classification methods for single-cell RNA-sequencing data. Brief Bioinform. 2020;21(4):1196–208. doi: 10.1093/bib/bbz062 31271412 PMC7444317

[9] Qi R, Liu S, Hui X, Shaytan AK, Liu B. AI in drug development: advances in response, combination therapy, repositioning, and molecular design. Sci China Inf Sci. 2025;68(7). doi: 10.1007/s11432-024-4461-0

[10] Qi R, Zou Q. Trends and Potential of Machine Learning and Deep Learning in Drug Study at Single-Cell Level. Research (Wash D C). 2023;6:0050. doi: 10.34133/research.0050 36930772 PMC10013796

[11] Frank R, Hargreaves R. Clinical biomarkers in drug discovery and development. Nat Rev Drug Discov. 2003;2(7):566–80. doi: 10.1038/nrd1130 12838269

[12] Breiman L. Random Forests. Machine Learning. 2001;45(1):5–32. doi: 10.1023/a:1010933404324

[13] Chen T. Xgboost: A scalable tree boosting system. Cornell University. 2016.

[14] Drucker H, Burges CJC, Kaufman L, Smola A, Vapoik V. Support vector regression machines. Advances in Neural Information Processing Systems. 1996;9.

[15] Ali M, Aittokallio T. Machine learning and feature selection for drug response prediction in precision oncology applications. Biophys Rev. 2019;11(1):31–9. doi: 10.1007/s12551-018-0446-z 30097794 PMC6381361

[16] Adam G, Rampášek L, Safikhani Z, Smirnov P, Haibe-Kains B, Goldenberg A. Machine learning approaches to drug response prediction: challenges and recent progress. NPJ Precis Oncol. 2020;4:19. doi: 10.1038/s41698-020-0122-1 32566759 PMC7296033

[17] Kearnes S, McCloskey K, Berndl M, Pande V, Riley P. Molecular graph convolutions: moving beyond fingerprints. J Comput Aided Mol Des. 2016;30(8):595–608. doi: 10.1007/s10822-016-9938-8 27558503 PMC5028207

[18] Hajim WI, Zainudin S, Mohd Daud K, Alheeti K. Optimized models and deep learning methods for drug response prediction in cancer treatments: a review. PeerJ Comput Sci. 2024;10:e1903. doi: 10.7717/peerj-cs.1903 38660174 PMC11042005

[19] Zulfiqar H, Guo Z, Ahmad RM, Ahmed Z, Cai P, Chen X, et al. Deep-STP: a deep learning-based approach to predict snake toxin proteins by using word embeddings. Front Med (Lausanne). 2024;10:1291352. doi: 10.3389/fmed.2023.1291352 38298505 PMC10829051

[20] Yan K, Lv H, Shao J, Chen S, Liu B. TPpred-SC: multi-functional therapeutic peptide prediction based on multi-label supervised contrastive learning. Sci China Inf Sci. 2024;67(11). doi: 10.1007/s11432-024-4147-8

[21] Wysocka M, Wysocki O, Zufferey M, Landers D, Freitas A. A systematic review of biologically-informed deep learning models for cancer: fundamental trends for encoding and interpreting oncology data. BMC Bioinformatics. 2023;24(1):198. doi: 10.1186/s12859-023-05262-8 37189058 PMC10186658

[22] Fernández-Torras A, Comajuncosa-Creus A, Duran-Frigola M, Aloy P. Connecting chemistry and biology through molecular descriptors. Curr Opin Chem Biol. 2022;66:102090. doi: 10.1016/j.cbpa.2021.09.001 34626922

[23] Wang Y, Zhai Y, Ding Y, Zou Q. SBSM-Pro: support bio-sequence machine for proteins. Sci China Inf Sci. 2024;67(11). doi: 10.1007/s11432-024-4171-9

[24] Yang Y, Gao D, Xie X, Qin J, Li J, Lin H, et al. DeepIDC: A Prediction Framework of Injectable Drug Combination Based on Heterogeneous Information and Deep Learning. Clin Pharmacokinet. 2022;61(12):1749–59. 10.1007/s40262-022-01180-9 36369328

[25] Sartori F, Codicè F, Caranzano I, Rollo C, Birolo G, Fariselli P, et al. A Comprehensive Review of Deep Learning Applications with Multi-Omics Data in Cancer Research. Genes (Basel). 2025;16(6):648. doi: 10.3390/genes16060648 40565540 PMC12191839

[26] Seal DB, Das V, Goswami S, De RK. Estimating gene expression from DNA methylation and copy number variation: A deep learning regression model for multi-omics integration. Genomics. 2020;112(4):2833–41. doi: 10.1016/j.ygeno.2020.03.021 32234433

[27] Wei L, Liao M, Gao Y, Ji R, He Z, Zou Q. Improved and Promising Identification of Human MicroRNAs by Incorporating a High-Quality Negative Set. IEEE/ACM Trans Comput Biol Bioinform. 2014;11(1):192–201. doi: 10.1109/TCBB.2013.146 26355518

[28] Chang Y, Park H, Yang H-J, Lee S, Lee K-Y, Kim TS, et al. Cancer Drug Response Profile scan (CDRscan): A Deep Learning Model That Predicts Drug Effectiveness from Cancer Genomic Signature. Sci Rep. 2018;8(1):8857. doi: 10.1038/s41598-018-27214-6 29891981 PMC5996063

[29] Liu P, Li H, Li S, Leung K-S. Improving prediction of phenotypic drug response on cancer cell lines using deep convolutional network. BMC Bioinformatics. 2019;20(1):408. doi: 10.1186/s12859-019-2910-6 31357929 PMC6664725

[30] Sharifi-Noghabi H, Zolotareva O, Collins CC, Ester M. MOLI: multi-omics late integration with deep neural networks for drug response prediction. Bioinformatics. 2019;35(14):i501–9. doi: 10.1093/bioinformatics/btz318 31510700 PMC6612815

[31] LeCun Y, Bengio Y, Hinton G. Deep learning. Nature. 2015;521(7553):436–44. doi: 10.1038/nature14539 26017442

[32] Kipf TN, Welling M. Semi-supervised classification with graph convolutional networks. arXiv preprint. 2016. doi: 10.48550/arXiv.1609.02907

[33] Liu Q, Hu Z, Jiang R, Zhou M. DeepCDR: a hybrid graph convolutional network for predicting cancer drug response. Bioinformatics. 2020;36(Suppl_2):i911–8. doi: 10.1093/bioinformatics/btaa822 33381841

[34] Nguyen T, Nguyen GTT, Nguyen T, Le D-H. Graph Convolutional Networks for Drug Response Prediction. IEEE/ACM Trans Comput Biol Bioinform. 2022;19(1):146–54. doi: 10.1109/TCBB.2021.3060430 33606633

[35] Huang Z, Fan Z, Shen S, Wu M, Deng L. MolMVC: Enhancing molecular representations for drug-related tasks through multi-view contrastive learning. Bioinformatics. 2024;40(Suppl 2):ii190–7. doi: 10.1093/bioinformatics/btae386 39230706 PMC11373324

[36] Jiang L, Jiang C, Yu X, Fu R, Jin S, Liu X. DeepTTA: a transformer-based model for predicting cancer drug response. Brief Bioinform. 2022;23(3):bbac100. doi: 10.1093/bib/bbac100 35348595

[37] Vaswani A, et al. Attention is all you need. Advances in neural information processing systems. 2017;30.

[38] Lao C, Zheng P, Chen H, Liu Q, An F, Li Z. DeepAEG: a model for predicting cancer drug response based on data enhancement and edge-collaborative update strategies. BMC Bioinformatics. 2024;25(1):105. doi: 10.1186/s12859-024-05723-8 38461284 PMC10925015

[39] Li X, Shi X, Li Y, Wang L. MCMVDRP: a multi-channel multi-view deep learning framework for cancer drug response prediction. J Integr Bioinform. 2024;21(3):20240026. doi: 10.1515/jib-2024-0026 39238451 PMC11602226

[40] Chen Y, Wang Z, Wang J, Chu Y, Zhang Q, Li ZA, et al. Self-supervised learning in drug discovery. Sci China Inf Sci. 2025;68(7). doi: 10.1007/s11432-024-4453-4

[41] Oloulade BM, Gao J, Chen J, Al-Sabri R, Wu Z. Cancer drug response prediction with surrogate modeling-based graph neural architecture search. Bioinformatics. 2023;39(8):btad478. doi: 10.1093/bioinformatics/btad478 37555809 PMC10432359

[42] Weininger D. SMILES, a chemical language and information system. 1. Introduction to methodology and encoding rules. J Chem Inf Comput Sci. 1988;28(1):31–6. doi: 10.1021/ci00057a005

[43] Min E, Chen R, Bian Y, Xu T. Transformer for graphs: An overview from architecture perspective. arXiv preprint. 2022. doi: arXiv:2202.08455

[44] Chu T, Nguyen TT, Hai BD, Nguyen QH, Nguyen T. Graph Transformer for Drug Response Prediction. IEEE/ACM Trans Comput Biol Bioinform. 2023;20(2):1065–72. doi: 10.1109/TCBB.2022.3206888 36107906

[45] Ying C, Cai T, Luo S, Zheng S, Ke G, He D, et al. Do transformers really perform badly for graph representation? Advances in Neural Information Processing Systems. 2021;34:28877–88.

[46] Tian Y, Krishnan D, Isola P. Contrastive Multiview Coding.

[47] Liang C, Wang D, Zhang H, Zhang S, Guo F. Robust Tensor Subspace Learning for Incomplete Multi-View Clustering. IEEE Trans Knowl Data Eng. 2024;36(11):6934–48. doi: 10.1109/tkde.2024.3399707

[48] Pearson K. VII. Note on regression and inheritance in the case of two parents. Proceedings of the Royal Society of London. 1895;58(347–352):240–2. doi: 10.1098/rspl.1895.0041

[49] Spearman C. The proof and measurement of association between two things. 1961. 10.1093/ije/dyq191 21051364

[50] Hyndman RJ, Koehler AB. Another look at measures of forecast accuracy. International Journal of Forecasting. 2006;22(4):679–88. doi: 10.1016/j.ijforecast.2006.03.001

[51] Mattingly CJ, Colby GT, Forrest JN, Boyer JL. The Comparative Toxicogenomics Database (CTD). Environ Health Perspect. 2003;111(6):793–5. doi: 10.1289/ehp.6028 12760826 PMC1241500

[52] Daina A, Michielin O, Zoete V. SwissTargetPrediction: updated data and new features for efficient prediction of protein targets of small molecules. Nucleic Acids Res. 2019;47(W1):W357–64. doi: 10.1093/nar/gkz382 31106366 PMC6602486

[53] Liu X, Ouyang S, Yu B, Liu Y, Huang K, Gong J, et al. PharmMapper server: a web server for potential drug target identification using pharmacophore mapping approach. Nucleic Acids Res. 2010;38(Web Server issue):W609-14. doi: 10.1093/nar/gkq300 20430828 PMC2896160

[54] Ashburner M, Ball CA, Blake JA, Botstein D, Butler H, Cherry JM, et al. Gene Ontology: tool for the unification of biology. Nat Genet. 2000;25(1):25–9. doi: 10.1038/75556 PMC3037419 10802651

[55] Liu H, Zhang B, Sun Z. Spectrum of EGFR aberrations and potential clinical implications: insights from integrative pan-cancer analysis. Cancer Commun (Lond). 2020;40(1):43–59. doi: 10.1002/cac2.12005 32067422 PMC7163653

[56] Levantini E, Maroni G, Del Re M, Tenen DG. EGFR signaling pathway as therapeutic target in human cancers. Semin Cancer Biol. 2022;85:253–75. doi: 10.1016/j.semcancer.2022.04.002 35427766

[57] Wang L, Liu W-Q, Broussy S, Han B, Fang H. Recent advances of anti-angiogenic inhibitors targeting VEGF/VEGFR axis. Front Pharmacol. 2024;14:1307860. doi: 10.3389/fphar.2023.1307860 38239196 PMC10794590

[58] Mosca E, Szigeti F, Tragianni S, Gallagher D, Groh G. SHAP-based explanation methods: a review for NLP interpretability. In: Proceedings of the 29th International Conference on Computational Linguistics, 2022.

[59] Sondka Z, Bamford S, Cole CG, Ward SA, Dunham I, Forbes SA. The COSMIC Cancer Gene Census: describing genetic dysfunction across all human cancers. Nat Rev Cancer. 2018;18(11):696–705. doi: 10.1038/s41568-018-0060-1 30293088 PMC6450507

[60] Huang Y, Fang Y, Wu J, Dziadyk JM, Zhu X, Sui M, et al. Regulation of Vinca alkaloid-induced apoptosis by NF-kappaB/IkappaB pathway in human tumor cells. Mol Cancer Ther. 2004;3(3):271–7. doi: 10.1158/1535-7163.271.3.3 15026547

[61] Ngoi NY, Pilié PG, McGrail DJ, Zimmermann M, Schlacher K, Yap TA. Targeting ATR in patients with cancer. Nature Reviews Clinical Oncology. 2024;21(4):278–93. 10.1038/s41571-024-00863-5 38378898

[62] Priyadarshini K, Keerthi AU. Paclitaxel against cancer: a short review. Med Chem. 2012;2(7):139–41.

[63] Yang W, Soares J, Greninger P, Edelman EJ, Lightfoot H, Forbes S, et al. Genomics of Drug Sensitivity in Cancer (GDSC): a resource for therapeutic biomarker discovery in cancer cells. Nucleic Acids Res. 2013;41(Database issue):D955-61. doi: 10.1093/nar/gks1111 23180760 PMC3531057

[64] Barretina J, Caponigro G, Stransky N, Venkatesan K, Margolin AA, Kim S, et al. The Cancer Cell Line Encyclopedia enables predictive modelling of anticancer drug sensitivity. Nature. 2012;483(7391):603–7. doi: 10.1038/nature11003 22460905 PMC3320027

[65] Kim S, Chen J, Cheng T, Gindulyte A, He J, He S, et al. PubChem 2019 update: improved access to chemical data. Nucleic Acids Res. 2019;47(D1):D1102–9. doi: 10.1093/nar/gky1033 30371825 PMC6324075

[66] Tsherniak A, Vazquez F, Montgomery PG, Weir BA, Kryukov G, Cowley GS, et al. Defining a Cancer Dependency Map. Cell. 2017;170(3):564-576.e16. doi: 10.1016/j.cell.2017.06.010 28753430 PMC5667678

[67] Landrum G. Rdkit documentation. Release. 2013;1(1–79):4.

[68] Siddiqui B, Yadav CS, Akil M, Faiyyaz M, Khan AR, Ahmad N, et al. Artificial Intelligence in Computer-Aided Drug Design (CADD) Tools for the Finding of Potent Biologically Active Small Molecules: Traditional to Modern Approach. Comb Chem High Throughput Screen. 2026;29(6):962–90. doi: 10.2174/0113862073334062241015043343 39819404

[69] Oord A v d, Li Y, Vinyals O. Representation learning with contrastive predictive coding. arXiv preprint. 2018. doi: 10.48550/arXiv.1807.03748

[70] Weisstein EW. Floyd-warshall algorithm. 2008. https://mathworld.wolfram.com/

